# Will Widespread Synthetic Opioid Consumption Induce Epigenetic Consequences in Future Generations?

**DOI:** 10.3389/fphar.2018.00702

**Published:** 2018-07-03

**Authors:** Federica Gilardi, Marc Augsburger, Aurelien Thomas

**Affiliations:** ^1^Forensic Toxicology and Chemistry Unit, University Center of Legal Medicine, Lausanne University Hospital – Geneva University Hospitals, Geneva, Switzerland; ^2^Faculty of Biology and Medicine, University of Lausanne, Lausanne, Switzerland

**Keywords:** opioids, transgenerational inheritance, epigenetics, parental exposure, prenatal exposure, vulnerability

## Abstract

A growing number of evidence demonstrates that ancestral exposure to xenobiotics (pollutants, drugs of abuse, etc.) can perturb the physiology and behavior of descendants. Both maternal and paternal transmission of phenotype across generations has been proved, demonstrating that parental drug history may have significant implications for subsequent generations. In the last years, the burden of novel synthetic opioid (NSO) consumption, due to increased medical prescription of pain medications and to easier accessibility of these substances on illegal market, is raising new questions first in term of public health, but also about the consequences of the parental use of these drugs on future generations. Besides being associated to the neonatal abstinence syndrome, *in utero* exposure to opioids has an impact on neuronal development with long-term repercussions that are potentially transmitted to subsequent generations. In addition, recent reports suggest that opioid use even before conception influences the reactivity to opioids of the progeny and the following generations, likely through epigenetic mechanisms. This review describes the current knowledge about the transgenerational effects of opioid consumption. We summarize the preclinical and clinical findings showing the implications for the subsequent generations of parental exposure to opioids earlier in life. Limitations of the existing data on NSOs and new perspectives of the research are also discussed, as well as clinical and forensic consequences.

## Introduction

The last decade is witnessing a huge increase in medical use and abuse of opioids, which is emerging as a major public health threat due to the concomitant dramatic rise in overdose morbidity and mortality (Humphreys, 2017; Kertesz, 2017). In the United States, epidemiological data indicate that the number of deaths involving opioids has more than quadrupled since 1999 (CDC, 2017), and the trend shows no sign of diminishing. The increase of opioid prescriptions to manage acute and chronic pain obviously contributed to generate this burden (Bedson et al., 2013; McCabe et al., 2017). In addition, opioid spread has been strongly favored by the easy accessibility of a number of licit (pharmaceutical or counterfeit), and illicit opioids of synthesis, cheaply manufactured on industrial scale and distributed online (Pergolizzi et al., 2018). These opioids include fentanyl, firstly synthetized in 1960 and approved as anesthetic and for palliative use, fentanyl analogs and novel synthetic opioids (NSOs), such as AH-7921, U-47700 and MT-45 butyrylfentanyl (Armenian et al., 2017). Newer compounds are also produced by clandestine manufacturers at a fast pace, which makes difficult their analytical detection and legal regulation by international drug agencies (Armenian et al., 2017). Most of these molecules are potent agonists of the μ-opioid receptor, while they are less active on the κ and δ isoforms. Opioid receptors are distributed throughout the central nervous system and mediate the analgesic, but also the adverse effects including respiratory depression, constipation, rewarding properties, etc. (Cox, 2011; Pasternak and Pan, 2013). Notably, increasing evidence suggests that, besides its direct effect on treated individuals, drug exposure may induce lasting effects on subsequent generations (Vassoler et al., 2014). Nevertheless, information about the possible transgenerational consequences of opioid use is still very limited, with relevant consequences at regulatory level for prescription. This review summarizes the molecular mechanisms that underlie transgenerational inheritance of drug exposure and the available data on opioids, focusing on human data. Due to the scarcity of studies specifically addressing NSOs, data on opiates and opioids will also be included.

## Molecular Mechanisms Underlying the Impact of Drugs on Future Generations

A family history of drug abuse correlates with increased risk of drug use in offspring (Yohn et al., 2015). However, only a small number of gene variants has been associated to drug addiction, indicating that genetics cannot provide the sole explanation. Indeed, environmental components, including drug consumption, may also influence the physiology and behavior of future descendants. The first demonstration of such impact referred to exposure to vinclozin, an agricultural fungicide, which can generate stable and heritable changes across several generations (Anway et al., 2005). Since then, many examples of both maternal and paternal phenotype transmission have been documented following prenatal stress (Morgan and Bale, 2011), diet variations (Kaati et al., 2002; Dunn and Bale, 2009; Champagne, 2010; Ng et al., 2010; Ost et al., 2014) and drug use/abuse (He et al., 2006; Novikova et al., 2008; Vassoler et al., 2014). In most cases, such transgenerational effects are mediated by epigenetic mechanisms. Epigenetics refers to all the molecular processes that regulate genome activity without changes in the DNA sequence (Skinner, 2011), which underlie, for instance, the ability of the same genome to produce multiple differentiated cell types in the same organism. Of note, epigenetic information responds to short- and long-term environmental inputs, allowing cells to adapt to new conditions, and such changes might be preserved during mitosis (Campos et al., 2014). Therefore, epigenetic remodeling events occurring in the germline can potentially persist through several generations, thus promoting effects also on individuals that were not exposed to the initial insult (Sharma and Rando, 2017).

**FIGURE 1:**
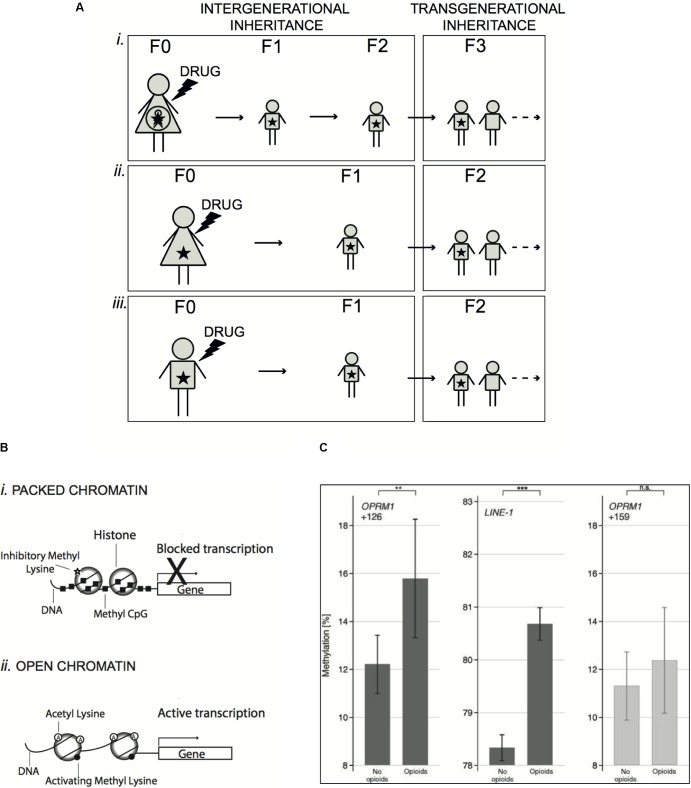
Mechanisms of epigenetic inheritance induced by drug exposure. **(A)** Exposure of a pregnant female (F0) to a drug (prenatal exposure) implies also exposure *in utero* of the fetus and its own future germline (i). In this case, inherited epigenetic effects rising in the newborn (F1) and the direct descendants (F2) are considered as intergenerational, because cells of the future organism were directly exposed. Only epigenetic changes preserved in the following generations (F3 and after) are transgenerational, as manifesting in the absence of any exposure. On the other hand, exposure can occur in females (ii) or males (iii) prior to pregnancy (parental exposure), thus potentially touching the germline, which will produce the next generation (F1). In this case, inherited epigenetic alterations will be considered as transgenerational already in the F2 generation and beyond. **(B)** Schematic representation of epigenetic mechanisms. In chromatin, DNA is wrapped around individual histone proteins. DNA methylation at CpG dinucleotides (Methyl CpG) and methylation of histones at specific lysine residues are associated to a condensed chromatin structure, where DNA is less accessible to transcription factors, which results in silenced transcription (i). In contrast, acetylation of histones and specific histone methylation favors a more open structure of the chromatin, which allows the recruitment of transcription factors and activation of transcription (ii). **(C)** DNA methylation (means and 95% confidence intervals) at *OPRM1* gene (position +126 counted from the adenine of the start codon, left panel) and LINE-1 (long-interspersed nuclear elements – central panel) in a cohort of 132 chronic pain patients of whom 62 were treated with opioid analgesics for more than 1 year. Methylation was higher in the opioid-treated patients than in age-matched non-opioid-treated pain patients. The significances (^∗∗^*P* < 0.01, ^∗∗∗^*P* < 0.001) are the results of *t*-test comparisons between groups. For comparison of the specificity of the hypermethylation at position +126 of the *OPRM1* gene, a non-significant position (+159) is shown at right. Data shown in C panel are from Doehring et al. (2013) (License No. 4331930562761).

Speaking about epigenetic inheritance, one important distinction relates to the type of exposure that can be **prenatal,** when occurring in a pregnant female or **parental,** if occurring prior to pregnancy (**Figure 1A**). In the first case, the possible effects are considered as true transgenerational inheritance, manifesting in the absence of any exposure, only if they are preserved at least in the third generation of descendants (F3) (Heard and Martienssen, 2014). By contrast, in case of parental exposure, we speak about transgenerational epigenetic alterations starting already in the F2 generation.

### Epigenetic Mechanisms

Epigenetic changes are able to regulate the expression of specific genes by remodeling the structure of chromatin thus enabling the transition from open and transcriptionally active state to a condensed and transcriptionally repressed state (Margueron and Reinberg, 2010). At molecular level, the modifications involved in epigenetic inheritance include mainly post-translational modifications (PTMs) of histones and DNA methylation, but also non-coding and coding RNAs (Heard and Martienssen, 2014).

Histone N-terminal tails are targets of a number of covalent, but reversible modifications, such as acetylation, methylation, phosphorylation, crotonylation, succinylation, ubiquitylation, citrullination, and O-GlcNAcylation (Allis and Jenuwein, 2016; Sharma and Rando, 2017). The transcriptional effects of distinct histone PTMs are different (**Figure 1B**). While histone acetylation is associated with transcriptional activation, histone methylation is implicated in both activation and repression of transcription, depending on the residue involved and on the level of methylation (Teperino et al., 2010). In mammals, sperm alterations of histone H3 acetylation and methylation were reported in response to cocaine (Vassoler et al., 2013), hepatotoxin (Gapp et al., 2014), and low-protein diet (Carone et al., 2010), although it is still unclear if these PTMs are sufficient to convey instructive information for the progeny.

Another fundamental epigenetic process is DNA methylation, which occurs typically on the cytosine of CpG dinucleotides, enriched in the proximity of gene promoters and enhancers (**Figure 1B**) (Margueron and Reinberg, 2010). DNA methylation is mostly associated with transcriptional suppression and this mechanism underlies several examples of genomic regulation, such as genomic imprinting (genes whose expression is determined only by the paternal or maternal allele), X-chromosome inactivation and epigenetic memory maintenance (Bergman and Cedar, 2013). In sperm, the degree of DNA methylation at various loci is influenced by environmental factors including diet (Radford et al., 2014), alcohol (Govorko et al., 2012) and traumatic stress (Gapp et al., 2014; Bohacek et al., 2015) and similar aberrations were observed in the brain of the offspring. However, also in this case, the demonstration that parental DNA methylation alterations are causally contributing to specific traits in the descendants is challenging, due to the difficulty to assign specific modifications to a given phenotype (Bohacek and Mansuy, 2015).

In mammals, most epigenetic changes arising in germline throughout life are actually erased during reproduction, which apparently leaves little chance for inheritance of epigenetic marks (Heard and Martienssen, 2014). Two reprogramming events of global DNA methylation take place in early embryonic development to promote cellular totipotency. However, at specific loci, some methylation and histone marks can escape this complete erasure (Bartolomei, 2009; Orozco et al., 2014; Sharma and Rando, 2017), which suggests that epigenetic modifications might be carriers of inheritable information. More recently, several reports described that sperm RNAs can convey the transfer of complex acquired phenotype from father to the offspring (Grandjean et al., 2015; Rodgers et al., 2015; Chen et al., 2016), likely through their ability to influence DNA methylation (Kiani et al., 2013). Although these findings pointed out a causal role of sperm RNAs in epigenetic germline inheritance, the underlying mechanisms remain unresolved so far.

## Consequences of Opioid Prenatal Exposure

According to recent reports, up to 1 in 5 women are taking an opioid medication at some point while pregnant (Desai et al., 2014). This is concerning because opioids are known to cross rapidly the placenta in concentrations consistent with maternal dose (de Castro et al., 2011) thus potentially triggering short and long-term vulnerabilities in the progeny. Prenatal opioid exposure can induce neonatal abstinence syndrome (NAS) in newborn infants, but knowledge about its long-term effects is limited, and information about possible transgenerational effects is even less abundant.

Neonatal abstinence syndrome is a true opioid withdrawal syndrome often requiring pharmacological treatment with replacement opioids and longer hospitalization to cope with symptoms including dehydration, diarrhea, fever, congestion, and diaphoresis (Practice and Medicine, 2017). A maintenance treatment with methadone or buprenorphine is the gold standard therapy for opioid-addicted pregnant women and NAS is estimated to occur in about 50% of infants chronically exposed to opioids (Klaman et al., 2017). This incidence corresponds to 5 out of 1000 live birth in United States (Patrick et al., 2015), with big health and economical implications, particularly because of the current inability to understand the factors associated to a severe NAS outcome. Indeed, despite multiple efforts aiming at modeling the contributions of maternal opioid dose and of the concurrent exposure to other medications or illicit drugs, the results remain so far inconclusive. Some genetic polymorphisms of genes related to dopamine and endogenous opioid systems such as prepronociceptin (*PNOC*) (Wachman et al., 2017), opioid receptors (Wachman et al., 2015) (*OPRM1*, *OPRK1*, and *OPRD1*), and catechol-*O*-methyltransferase (*COMT*) (Wachman et al., 2013), seem associated to a more severe NAS outcome, although further test on a larger scale are required to confirm these indications. Notably, one report showed that high methylation of three specific CpG sites of the *OPRM1* promoter is associated to a worse NAS outcome in newborn babies from mothers receiving methadone or buprenorphine during pregnancy, likely due to the subsequent lower expression of the receptor and a need for higher doses of opioid medication to control NAS symptoms (Wachman et al., 2014).

Regarding the long-term consequences of *in utero* opioid exposure, clinical studies in humans are extremely complicated by the huge amount of variables (i.e., doses and length of treatment) and of concurring risk factors that are often present, such as polysubstance use, stability, mother–child interaction, etc. Animal studies, performed mostly in rodents and in rigorously controlled experimental conditions, have helped to partially fill this gap. These studies highlighted broad neurodevelopmental effects of prenatal opioid exposure, including long-lasting changes in pre- and post-synaptic activity, altered opioid-mediated analgesia, reward-related behaviors, and impairment of hippocampal-based learning, in addition to alterations of the immune response (for recent review readers can refer to Byrnes and Vassoler, 2017). Unfortunately, these investigations almost completely referred to morphine, with only few exceptions examining oxycodone (Davis et al., 2010; Devarapalli et al., 2016), methadone (Hou et al., 2004; Vestal-Laborde et al., 2014; Wong et al., 2014; Chiang et al., 2015), and buprenorphine (Hung et al., 2013; Chiang et al., 2014; Wu et al., 2014). To the best of our knowledge, no report exists on long-term effects of prenatal administration of other synthetic opioids, such as fentanyl, in animal models. In addition, the heritability of such changes in the following generations was not really investigated. Alarmingly, in spite of converging animal data indicating possible long-term consequence of prenatal exposure to opioids, only few studies addressed the fate of exposed infants as they grow and enter adolescence and young adulthood. Young children born from women exposed to opioids during pregnancy show increased likelihood of problems related to motor skills, attention, and behavior regulation (Ornoy et al., 2001; Slinning, 2004; Melinder et al., 2013; Sundelin Wahlsten and Sarman, 2013). More divergent findings are reported concerning general cognitive abilities, with some study indicating an impairment of memory abilities in exposed children (Bunikowski et al., 1998; Hunt et al., 2008; Salo et al., 2009; Sundelin Wahlsten and Sarman, 2013), whereas others show no differences (Rosen and Johnson, 1985; de Cubas and Field, 1993; Melinder et al., 2013). Less information is available about adult offspring of opioid-dependent users, although few longitudinal studies reported deficits on several cognitive parameters (Konijnenberg et al., 2016; Nygaard et al., 2016, 2017). Collectively, however, these data must be taken with caution due to the heterogeneity of prenatal drug exposure and the difficulty to dissociate opioid effects from other risk factors to which they are often associated.

It is important to mention that, although most available studies refer to infants/young adult born to opioid-dependent women, many other patients are prescribed opioids during the pregnancy for pain control issues [severe migraine headache, myalgia, joint pain, low back, and pelvic pain (Bateman et al., 2014)]. A report referring to more than 1 million pregnant women with low socioeconomical status in the United States, highlighted that 21.6% was dispensed at least once with prescription opioids during pregnancy and a significant increase was observed between the beginning (2000) and the end (2007) of the enrolment (Desai et al., 2014). The percentage was slightly lower, but still substantial (14%) in more affluent women (commercially insured) (Bateman et al., 2014). In Europe, data collected from a population-based registry covering the entire Norwegian population showed that, between 2004 and 2006, 6% of the pregnant women who ended the pregnancy filled at least one opioid prescription (Engeland et al., 2008). Chronic treatment with prescription opioids seems less diffused, as reported by a retrospective study on the period between 1998 and 2009, which recorded opioid use for more than 1 month during pregnancy in 6 out of 1000 deliveries (Kellogg et al., 2011). Regarding their prescription in pregnancy, most opioids were classified by the Food and Drug Administration under category C (**Table 1**), indicating that animal studies provided evidence for potential harm to the fetus, but human studies are lacking. Considered as overly simplistic, letter pregnancy categories were removed from drug labeling in 2015 and now risks for drug use during pregnancy, breast-feeding and in females and males of reproductive potential must be detailed. However, it remains difficult to infer from the available data if and at which doses/treatment conditions the use of opioids in pregnancy is safe. A comprehensive study highlighted an association between medical use of opioids in the first trimester of pregnancy and heart and neural tube birth defects (Interrante et al., 2017), while others refer to the third trimester of pregnancy (Coluzzi et al., 2014) and no study that we are aware of investigated the association with inheritable changes. Importantly, to counteract excessive opioid use, cannabinoids are emerging as alternative or combination treatment, due to the tight reciprocal interactions that exist between opioid and endocannabinoid signaling (Hurd, 2017). However, not even medical marijuana is devoid of risk of inducing hereditary effects (recently reviewed by Szutorisz and Hurd, 2018). Thus, it remains an urgent need of systematic longitudinal studies investigating the actual long-term impact of prenatal opioid exposure on the progeny and in subsequent generations.

**Table 1:** Classification of opioid medications according to the FDA pregnancy category rule valid until June 2015.

Active substance	Common names of medication	Use in pregnancy category (FDA)
Buprenorphine	Belbuca, Bunavail, Buprenex, Buprenorphine, Butrans, Probuphine, Sublocade, Suboxone, Zubsolv	C
Codeine	Butalbital, Carisoprodol, Fioricit, Codeine, Fioricet, Fiorinal, Prometh VC, Synalgos, Tylenol	C
Fentanyl	Abstral, Actiq, Duragesic, Fentanyl, Fentora, Ionsys, Lazanda, Sublimaze, Subsys	C
Hydrocodone	Anexsia, Apadaz, Flowtuss, Hycofenix, Hydrocodone bitartrate, Hysingla, Norco, Reprexain, Rezira, Tussicaps, Tussigon, Vicodin, Vituz, Zohydro ER, Zutripro	C
Methadone	Dolophine hydrochloride, methadone hydrocloride, Methadose	C
Morphine	Apokyn, Arymo ER, Astramorph PF, Duramorph PF, Embeda, Infumorph, Kadian, Morphabond ER, MS Contin	C
Oxycodone	Oxaydo, Oxycet, Oxycodone, Oxycodone hydrochloride, Oxycontin, Percocet, Percodan, Roxicet, Roxicodone, Roxybond, Xtampza ER	B
Tapentadol	Nucynta, Tapentadol Hydrochloride	C
Tramadol	Conzip, Tramadol Hydrochloride, Ultracet, Ultram	C

*Commercial medication names are listed in alphabetical order. ***Category B:*** no evidence of harm to the fetus in animal studies, but no adequate and well-controlled studies in pregnant women. ***Category C:*** some evidence of adverse effect in animal studies OR no animal studies have been conducted AND no adequate and well-controlled studies in pregnant women.*

## Inheritance Linked to Opioid Parental Exposure

As mentioned above, epigenetic changes induced by drug exposure in the germline might be inherited by descendants. In term of public health, the potential ability of opioids to trigger transgenerational effects following drug exposure before pregnancy might generate considerable long-term consequences in the population. The μ-opioid receptor is expressed in sperm cells and β-endorfin, an endogenous opioid, is produced locally in male reproductive tract (Albrizio et al., 2006). Expression of all opioid receptors is also detected in oocytes and, interestingly, the pattern of μ- and κ-opioid receptors is changing during oocyte maturation, which points to a possible role of endorphins in this process (Agirregoitia et al., 2012). The presence of opioid receptors in both gamete types is suggestive not only of a contribution of endorphins to maintain gamete function, but also of possible epigenetic effects triggered by opioids on these cells that, in turn, could be transmitted to subsequent generations. Consistent with this hypothesis, opioid addiction increased DNA methylation at specific sites of the *OPRM* gene promoter in several cells, including sperm (Nielsen et al., 2009; Chorbov et al., 2011; Ebrahimi et al., 2018). Interestingly, an *in vitro* study showed that morphine inhibits cellular cysteine uptake thus altering the redox state of the cells, which results in reduced availability of *S*-adenosyl methionine (SAM), the principal methyl donor for DNA methylation (Trivedi et al., 2014). Accordingly, global reduction of DNA methylation was observed in cells treated with morphine, while an opposite effect was found in leukocytes of chronic pain patients treated with opioid analgesics (**Figure 1C**) (Doehring et al., 2013). The apparent lack of congruence of these results might be explained by cell specific responses induced by opioids. Nevertheless, these reports unequivocally demonstrate that opioids induce epigenetic changes. Accordingly, in mice, morphine reduced histone methylation (Sun et al., 2012) and augmented histone H3 acetylation in nucleus accumbens (Sheng et al., 2011) and basolateral amigdala (Wang et al., 2015), two brain regions involved in the reward control. In humans, most reports highlighted opioid-induced epigenetic alterations only in the exposed generation, where they can mediate some of the observed behavioral effects, while there is not direct evidence of their transmission to subsequent generations. However, several studies in animals have started to investigate the impact of parental exposure to morphine at adolescence (F0) to the offspring (F1). Typically, adolescent female rats were treated with morphine and, after a wash out period, mated with drug-naïve males and tests were performed in adult F1 generation. Both male and female F1 rats showed enhanced locomotor activity when parents were exposed to morphine (Byrnes, 2005). In addition, males exhibited a more rapid development of morphine tolerance (Byrnes et al., 2011) and attenuated locomotor sensitization in association to increased expression of the dopamine D2 and κ-opioid receptors in nucleus accumbens (Byrnes et al., 2013). In contrast, in F1 females, parental morphine exposure altered anxiety-like behaviors (Byrnes et al., 2011), increased the sensitivity to opioid rewarding effects, likely due to sex-specific induction of the μ-opioid receptor (Vassoler et al., 2016), and lowered the levels of morphine self-administration (Vassoler et al., 2017). Thus, parental exposure to morphine induces neuroadaptation in both dopamine and opioid signaling and reshapes drug response in a sex-dependent manner. Moreover, alterations in hippocampal synaptic plasticity, with possible consequence on memory performance, were highlighted in the offspring of either F1 male or female (Sarkaki et al., 2008). Beyond the effects described in F1 generation, the first evidence of true transgenerational inheritance of opioid-induced effects came for the observation that F2 offspring from F0 morphine-exposed fathers exhibits decreased expression of synaptophysin and reduced synaptic connection (Vyssotski, 2011). Of note, changes in drug seeking behavior and drug tolerance were also observed in F2 generation from females exposed at adolescence (Byrnes et al., 2013; Vassoler et al., 2017), indicating that even limited exposure to opioids can have lasting effects across multiple generations. Whether these transgenerational repercussions are limited to opioid exposure during adolescence, when the reproductive system is still maturing, or if they are also present when exposure occurs in adults, remains to be verified.

## Conclusion

Medical use and misuse of opioids have strongly increased in the last decades and the consequences for public health are numerous. Besides the main effects on the directly exposed individuals, converging evidence suggests that opioids can induce long-lasting transgenerational changes in subsequent generations, particularly concerning drug sensitivity and tolerance, with possible implications for drug abuse vulnerability. However, both preclinical and clinical studies are currently too limited to draw rigorous conclusions on the actual impact that the spread NSO use might have on future generations. One big limitation relies on data mostly referring to a small group of molecules such as morphine, methadone and buprenorphine, whose relevance is restricted to specific conditions (i.e., replacement therapy during pregnancy), while a gap of knowledge persists for other NSO medications used for pain control, also during pregnancy. Consequently, the doses of substances that can be considered as safe not only for the mother, but also for the child and future generation still remain an open question for lots of NSOs. Moreover, given the increasing alternative use of opioids together with cannabinoids, the study of possible effects of such combinations might be highly relevant. A second crucial point is the very limited amount of publications investigating the transmission across multiple generations of parental opioid exposure. Moreover, future studies should consider not only mother, but also father habits, as epigenetic transmission occurs also through paternal gametes. Another critical aspect depends on the complex interpretation of the clinical studies that tried to address opioid effects on the following generations, because of the co-occurrence of many confounding factors (polysubstance use, genetic component). Therefore, preclinical studies must be carefully designed to increase as much as possible the translational relevance of the results and help establishing cause-effect relationships and the role of epigenetics. For instance, all animal studies investigating the effects of parental exposure to morphine were performed in a similar experimental paradigm with exposure at adolescence. Thus, so far, we totally lack information about the potential transgenerational impact of opioid exposure at adulthood that corresponds to the age with major NSO consumption in humans.

In conclusion, a huge research effort is warranted to inform the regulatory measures that are needed to curb the spread of synthetic opioids and to keep the risk–benefit ratio of the medicinal use of opioids as low as possible.

## Author Contributions

FG wrote the manuscript. MA and AT conceived and edited the manuscript.

## Conflict of Interest Statement

The authors declare that the research was conducted in the absence of any commercial or financial relationships that could be construed as a potential conflict of interest.
